# Tumor necrosis factor-α G-308A (rs1800629) polymorphism and aggressive periodontitis susceptibility: a meta-analysis of 16 case-control studies

**DOI:** 10.1038/srep19099

**Published:** 2016-01-11

**Authors:** Xue-Mei Wei, Yong-Ji Chen, Lan Wu, Li-Jun Cui, Ding-Wei Hu, Xian-Tao Zeng

**Affiliations:** 1 Department of Nursing, Affiliated Hospital of North Sichuan Medical College, Nanchong 637000, P.R. China; 2 Department of Stomatology, Taihe Hospital, Hubei University of Medicine, Shiyan 442000, Hubei Province, China; 3 Department of Vascular Surgery, Affiliated Hospital of North Sichuan Medical College, Nanchong 637000, P.R. China; 4 Center for Evidence-Based and Translational Medicine, Zhongnan Hospital of Wuhan University, Wuhan 430071, China

## Abstract

Association between tumor necrosis factor-α (TNF-α) G-308A (rs1800629) polymorphism and susceptibility to aggressive periodontitis (AgP) were inconsistent, hence we performed this meta-analysis to clarify the association between them using Comprehensive Meta-Analysis v2.2 software. 16 case-control studies were searched from the PubMed, Embase and CNKI databases up to February 2, 2015. The meta-analysis showed a significantly increased risk in A vs. G (*OR* = 1.23, 95%*CI* = 1.04–1.44), AA vs. GG (*OR* = 2.07, 95%*CI* = 1.11–3.87), and AA vs. AG+GG genetic models (*OR* = 2.09, 95%*CI* = 1.13–3.86); however, the non-significantly increased risk was shown in AG vs. GG (*OR* = 1.06, 95%*CI* = 0.85–1.32) and AA+AG vs. GG genetic models (*OR* = 1.06, 95%*CI* = 0.85–1.31). Cumulative analysis showed that the association changed from non-significant to significant with new studies accumulated and the *CIs* became more and more narrow, sensitivity analysis indicated results were statistically robust. Stratified analyses of confirmed of HWE, Asians, Caucasians, and population-based controls obtained results similar to that of overall analysis. There was no evidence of publication bias. In summary, current evidence demonstrates that TNF-a G-308A polymorphism might be associated with AgP susceptibility, especially in Asians and Caucasians.

Aggressive periodontitis (AgP) is featured of the rapid rate of disease progression, onset in healthy individuals without large accumulation of plaque and/or calculus, and with genetic familial trait^1^,^2^,^3^. In 1999, the new term “aggressive periodontitis” have been proposed to replace the former nomenclature “early-onset periodontitis”, which contained “juvenile periodontitis”, “rapidly progressive periodontitis”, and “prepubertal periodontitis”^4^. Concerning the serious hazards of periodontal tissues of patients with AgP in adolescents, it has widely involved more and more scholars and etiological researches referred to the molecular biology, genetics, microbiology, cell biology and other fields for its complex etiology. Surely, some pathogens are the external initiating factor in the pathogenesis of AgP, and the degrees of harm risk are inconsistent, suggesting that host heterogeneity might be a decisive factor in the pathogenesis of AgP^5^. Hence, AgP can be considered as a complex genetic disease and the influence of genes and environmental factors determine the individual phenotype corporately^6^.

With the deepen research of human gene, the more extensive evidence of pathogenesis of periodontitis has been provided^7^. Researches indicated nearly half of the clinical differences of periodontal disease rooted from gene polymorphism^8^. Therefore, many gene polymorphisms have been investigated^9^,^10^,^11^,^12^,^13^. Tumor necrosis factor (TNF) - α is one of the most potent proinflammatory cytokines and play a role in tissue injury and induced bone resorption in the immune response system, and its coding gene has been mapped to chromosome 6. The G-308A (rs1800629) is a polymorphism causing a substitution from the guanine (G) to adenine (A) and leads to two- to three fold higher transcriptional activity of TNF-α upon stimulation with bacterial lipopolysaccharide^14^,^15^. Carriage of the rare -308 A allele is associated with significantly greater TNF-α production and transcription. In addition, the A allele has been associated with increased risk for various non-related infectious and inflammatory diseases, including periodontitis.

In 2013, Song *et al.* performed a meta-analysis and indicated that TNF-a -308 A allele was associated with periodontitis^10^; However, their meta-analysis pooled chronic periodontitis (CP) and AgP together and included only 6 case-control studies for AgP. Nowadays, there were 17 studies that explored the association between TNF-α G-308A polymorphism and AgP have been published. The results of these published studies remain inconsistent. Therefore, this meta-analysis was conducted to provide an updated approach on the overall relationship between TNF-α G-308A polymorphism and AgP. Subgroup analyses were also performed on smoking and non-smoking status to investigate smoking-specific effects and cumulative analysis was used to investigate the trend of association.

## Methods

We followed the recommended Preferred Reporting Items for Systematic Reviews and Meta-Analyses (PRISMA) statement^16^ to report this meta-analysis, and the ethnic review was not required.

### Eligibility criteria

According to the “PICOS” principle^16^, this meta-analysis included studies which met the following criteria: (i) evaluating the association between TNF-α G-308A polymorphism and AgP, or all periodontitis but the data for AgP could be extracted; (ii) the study design was a case-control or cohort; (iii) the publication language was Chinese or English, and the full-text can be obtained; (iv) the relevant data was detailed enough to be used in the calculation, or the odds ratio (*OR*) with its 95% confidence interval (*CI*) was reported; (v) when more than one publications covered the same study population, we only included the study with higher quality and more comprehensive information. Animal experiments, conference abstracts, and comments were excluded. Studies with AgP patients who had any systematic diseases, such as diabetes mellitus, coronary heart disease were also excluded.

### Search strategy

A systematical search to retrieve published literatures from PubMed, Embase, and CNKI (China Knowledge Resource Integrated) databases was conducted up to February 2, 2015. The following key words and subject terms were used: “tumor necrosis factor alpha”, “TNF-α”, “TNF-alpha”, “periodontitis”, “periodontal disease”, “polymorphism”, “mutation”, and “variant”. Hand-searching of listed references of included studies, previous meta-analysis, and recent reviews was also performed to identify additional studies.

### Data extraction

The process of the data extraction was independently conducted and cross checked by two authors, and a third author participated in the discussion in case of divergence. We extracted the information as follows: the first author and year of publication, study area, ethnicity, smoking status, genotyping methods, the source of the controls, Hardy-Weinberg equilibrium (HWE) of control, and numbers of eligible genotyped cases and controls with the exhaustive data of each genotype distribution or the *OR* with its 95%*CI*.

### Statistical analysis

The *OR* with its 95% *CI* in the allelic contrast (A vs. G), recessive model (AA vs. AG+GG), dominant model (AA+AG vs. GG), and co-dominant model (AA vs. GG and AG vs. GG) were calculated to assess the association between TNF-α G-308A polymorphism and AgP risk. First, heterogeneity among included studies was detected using *I*^2^ and Cochran *Q* statistics, and the value of *I*^2^ ≤50% and *p* > 0.1 indicates low heterogeneity and using fixed-effects model, otherwise the random-effects model is used^17^,^18^,^19^,^20^. We conducted stratification analyses on the bias of control conform to HWE, ethnicity, source of control, and smoking status. In order to investigate the robustness of overall analysis, we conducted sensitivity analysis by deleting each included study in turn^21^,^22^,^23^. The cumulative analysis was also carried out to explore the trend of association^24^,^25^. Moreover, we performed meta-regression to figure the influence of smoking status for TNF-α G-308A polymorphism and the susceptibility of AgP. Publication bias was analyzed by funnel plot and Egger’s test^26^. All the above mentioned statistical analyses were undertaken using the Comprehensive Meta-Analysis v2.2 software^21^,^22^,^23^.

## Results

### Characteristics of included studies

**Figure 1:**
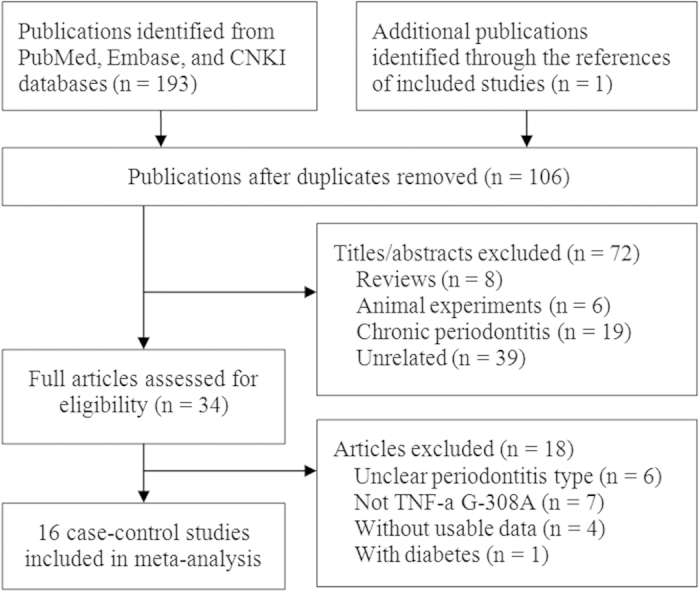
Study selection flow diagram.

A total of 194 citations was yielded and finally 16 case-control studies involving 905 cases and 1270 controls were included^27^,^28^,^29^,^30^,^31^,^32^,^33^,^34^,^35^,^36^,^37^,^38^,^39^,^40^,^41^,^42^. One was excluded due to the AgP patients with type 1 diabetes mellitus^43^. Figure 1 showed the process of study selection.

Seven studies both included smokers and non-smokers^30^,^31^,^33^,^34^,^35^,^37^,^41^, six only included non-smokers^29^,^32^,^36^,^38^,^40^,^42^, and three did not report smoking status^27^,^28^,^39^. One study only reported the *OR* and 95%*CI* of Allele comparison^39^, the others all reported the genotyping distribution. The controls of two studies out of HWE^30^,^42^. Detailed information of characteristics of included studies is available in Table 1.

### Overall, cumulative, and sensitivity analyses

**Figure 2:**
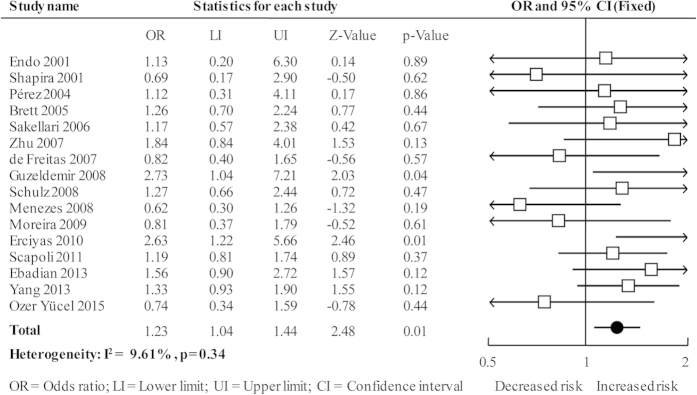
Forest plot of overall analysis in allele comparison.

The overall analysis showed a significantly increased risk in A vs. G (*OR* = 1.23, 95%*CI* = 1.04–1.44; Fig. 2), AA vs. GG (*OR* = 2.07, 95%*CI* = 1.11–3.87; see Supplementary Fig. S1 online), and AA vs. AG+GG genetic models (*OR* = 2.09, 95%*CI* = 1.13–3.86; see Supplementary Fig. S2 online); however, the non-significant increased risk was shown in AG vs. GG (*OR* = 1.06, 95%*CI* = 0.85–1.32; see Supplementary Fig. S3 online) and AA+AG vs. GG genetic models (*OR* = 1.06, 95%*CI* = 0.85–1.31; see Supplementary Fig. S4 online). The heterogeneity of all the five genetic models were acceptable and the results were pooled using fixed-effects model (Table 2).

**Figure 3:**
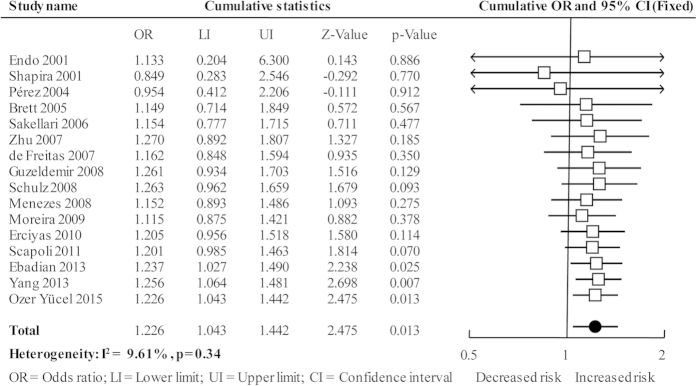
Cumulative analysis plot of overall analysis in allele comparison.

**Figure 4:**
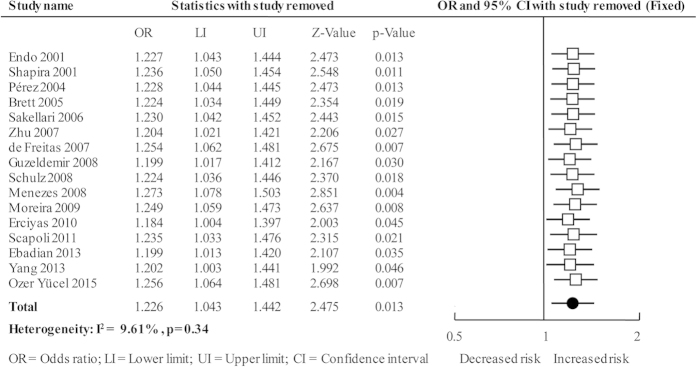
Sensitivity analysis plot of overall analysis in allele comparison.

The cumulative analysis was performed by accumulating included studies sequentially with increasing publication year, and the result showed that the association changed from non-significant to significant with new studies accumulated and the *CIs* became more and more narrow (Fig. 3). The sensitivity analysis was performed by deleting every included studies of this meta-analysis one by one, the results were not materially altered that indicating our results were statistically robust (Fig. 4).

### Subgroups, meta-regression, and publication bias analyses

Table 2 showed the results of all performed subgroup analyses. Stratified analyses of confirmed of HWE, Asians, Caucasians, and population-based controls obtained results similar to that of the overall analysis; however, the results of out of HWE studies, hospital-based controls, mixed smoking status, non-smokers, Brazilians, and Chileans all revealed non-significant association between TNF-α G-308A polymorphism and AgP. The results of meta-regression analysis showed that the smoking status can’t affect the association between TNF-α G-308A polymorphism and the susceptibility to AgP (see Supplementary Fig. S5 online).

**Figure 5:**
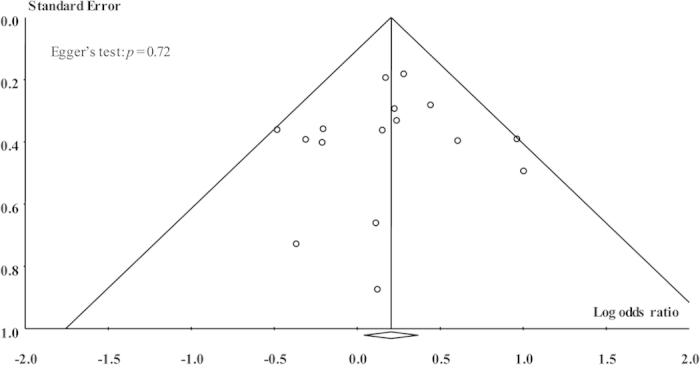
Funnel plot of overall analysis in allele comparison.

Dependably, no evidence of publication bias was found in this meta-analysis for any genetic models, which was supported by Egger’s test (A vs. G: *p* = 0.72; AA vs. GG: *p* = 0.99; AG vs. GG: *p* = 0.66; AA vs. AG+GG: *p* = 0.97; and AA+AG vs. GG: *p* = 0.25) and symmetric funnel plots (Fig. 5).

## Discussion

### Main findings

AgP, a distinguished subgroup of CP with the characteristics of familial aggregates, rapid disease progression and high incidence among period of adolescents, has a strong influence on the health of periodontal tissue. Epidemiologic studies showed that the incidence of AgP differed widely among different areas, different countries and different races. Unsatisfactorily, related reported dates were relative limited, ranging from 0.1% to 15% among overseas^44^. Now there are numerous studies on gene polymorphisms of inflammatory factor but haven’t come to an accordant conclusion^45^. As the local inflammation of the body, inflammatory factor TNF-α has an important stimulation and has a wide biological effect on leukocytes, vascular endothelium cells and different cells in connective tissue. TNF-α can cause the destruction of connective tissue and strengthen the formation and activity of osteoclast, thereby limiting the repair of periodontal tissue^46^. Our meta-analysis with 16 case-control studies quantitatively explores the relationship between TNF-α G-308A polymorphism and the susceptibility to AgP, demonstrating that the TNF-α G-308A polymorphism confers a weak susceptibility to AgP, and sensitivity analyses showed the results were robust. Subgroups analyses showed that TNF-a G-308A polymorphism might be associated with Caucasian and Asians, but not associated with Brazilians and Chileans. The results of cumulative analysis showed that the association changed from non-significant to significant with new studies accumulated, and this might indicate that statistical significance could be detected when sample size became enough.

Considering smoking is responsible for periodontitis to some certain degree^47^,^48^, we conducted a subgroup analysis based on smoking status. The results showed that the association between TNF-α G-308A polymorphism and the susceptibility to AgP was not affected by the smoking status in all contrast gene type, thus smoking status maybe not be a factors of special importance in TNF-α G-308A polymorphism, and this was also confirmed by meta-regression analysis. However, the results of HWE and source of controls indicated that studies out of HWE and studies with controls from hospital might bias the result.

### Strengths of study

The previous meta-analysis by Song *et al.*^10^ in 2013 investigated the TNF-α G-308A polymorphism and CP and AgP at the same time. This meta-analysis included 6 case-control studies for AgP and yielded negative association. Compared with CP, AgP refers to uncommon forms of bacterially-induced periodontitis and is considered as a genetically inherited disease^6^,^22^,^23^,^49^. Hence, our study is the first meta-analysis focusing on the association between TNF-α G-308A polymorphism and AgP risk. We also considered the influence of smoking status and stratified analysis according to the smoking status, which could provide more valuable information on this topic. Additionally, our meta-analysis included 16 case-control studies and the results are more precise, and the cumulative analyses also proved this. Moreover, we conducted four subgroups meta-analysis based on characteristics of studies and explained these roundly, while their just for race. Obviously, compared with this meta-analysis, our study carried out cumulative meta-analysis, smoking status based analysis and meta-regression analysis. Of course, our meta-analysis provided more advisory for periodontitis prevention, diagnosis, and treatment.

Our meta-analysis, conducted following a canonical systematically processes, for which some specific points need to be interpreted here. As we all know, the nomenclature of AgP has evolved with further understanding about the disease, but some researchers failed to capture the new terms in time, precisely, we not only took the present name “aggressive periodontitis, AgP” but also the former name “early-onset periodontitis, EOP” into account to ensure comprehensively covering eligible studies. The studies of Zhu *et al.* in 2007^33^ compared the differences of gene polymorphisms between male and female and reached the conclusion that TNF-α G-308A might be associated with the susceptibility to AgP for male individuals in China, while other included studies did not implement analysis by gender, accordingly the factor of genders cannot be analyzed here, but may be of value.

### Limitations of study

Meta-analysis is a secondary research, and the result of its researches largely depended on primary studies^50^, so there inevitably existed some limitations. First, 9 included studies were conducted based on Caucasian but for other races only 1 to 3 studies could be acquired, indicating limitations from the insufficient numbers of primary researches for some subgroups based on ethnicity; moreover, the sample sizes in some included studies, overall and subgroups analyses were relatively small. The limited sample sizes might bias the result. The cumulative analysis also indicated that the association became significant when the thirteen study accumulated. According to this, we must treat all results of subgroup analyses with caution. Second, it is hard for researchers to ensure strict consistency when they conduct research under the control of related factors criteria, such as age range, physical condition and lifestyle of the included populations, the diagnose index of AgP, the genotyping methods of included studies, which will affect the result of research to some degree. Of course, these are also the shortcomings of case-control study. Our meta-analysis based on case-control studies and did not escape from the influence of these factors. Third, publication bias test showed no evidence of publication bias existed, but this research conducted a comprehensive retrieval with language limited to Chinese and English, thus it may lead to the corresponding language bias, although we know this was limited by the accessibility to databases and ability of language. Fourth, the defect of a single gene may be a weak factor to promote the cause of disease directly for the normal function of proteins will not easily change that kind of greatly, so the combined polygenic factors are worth considering strengthening the evidence. Additionally, the adjusted data could not be extracted from included studies and we can only carry our analyses based on crude data. All in all, we should take the conclusion of this study seriously.

### Implications for practice and research

From the foregoing, as for the association between TNF-α G-308A polymorphism and AgP susceptibility, large-scale and high-quality researches can be implemented in point. Owing to the complex causes of AgP alone with a wide range of risk of factors, such as individual immunity, special microbial infection, heredity, certain diseases (e.g. diabetes), gender, habits (e.g. smoking, diet), stress, certain genetic polymorphisms, etc, future researches should try to strictly control various confounding factors to ensure the reliability of the results with explicit and strict inclusion criteria of cases. Thus, there are some points we recommend for further practices and researches. As we know, smoking is a risk factor and influence the treatment effect of periodontitis^48^,^51^, moreover, subgroup analysis and meta-regression analysis both indicated that this polymorphism was not influenced by smoking status (Table 2 and see Supplementary Fig. S5 online). This might mean that we could not know whether TNF-α G-308A polymorphism is a marker of smoking rather or a risk factor of AgP susceptibility. Hence, what the role of TNF-α G-308A polymorphism in the development of AgP is necessary to research either in smokers or non-smokers. Large-scale studies combining multiple loci might be of significance; then, further investigations based on the difference between population characterizes, such as gender, age, family history, can be developed; furthermore, combining with other type diseases or risk factors facilitated to AgP to explore the susceptibility between gene polymorphisms and AgP with multiple dangerous risk factors may be an additional direction of research. Moreover, from the implications of subgroups analysis and cumulative analysis, more relevant studies should be performed due to the small sample size currently. We also suggest that further studies should provide adjusted data, use population-based design, use uniformed AgP diagnosis criteria, and clearly reported the smoking status and ethnicity.

## Additional Information

**How to cite this article**: Wei, X.-M. *et al.* Tumor necrosis factor-a G-308A (rs1800629) polymorphism and aggressive periodontitis susceptibility: a meta-analysis of 16 case-control studies. *Sci. Rep.* **6**, 19099; doi: 10.1038/srep19099 (2016).

## Supplementary Material

Supplementary Information

## Figures and Tables

**Table 1:** Characteristics of studies included in the meta-analysis.

References	Country (ethnicity)	Smoking status	Genotyping method	Source of control	Case/ Control			HWE
					Sample	G	A	
Endo 2001	Japan (Asian)	Unknown	PCR-SSOP	PB	46/104	90/204	2/4	Yes
Shapira 2001	Israel (Caucasian)	Unknown	PCR-TaqMan	PB	16/27	29/47	3/7	Yes
Pérez 2004	Chile (Chileans)	Non-smokers	PCR-RFLP	PB	27/30	49/55	5/5	Yes
Brett 2005	UK (Caucasian)	Mixed	PCR-SSP	PB	50/97	76/155	24/39	No
Sakellari 2006	Greek (Caucasian)	Mixed	PCR	PB	46/90	78/156	14/24	Yes
Zhu 2007	China (Asian)	Mixed	PCR-RFLP	HB	64/78	111/144	17/12	Yes
de Freitas 2007	Brazil (Caucasian)	Non-smokers	PCR-RFLP	PB	30/70	46/102	14/38	Yes
Guzeldemir 2008	Turkey (Caucasian)	Non-smokers	PCR	PB	31/31	46/55	16/7	Yes
Schulz 2008	Germany (Caucasian)	Mixed	PCR	PB	69/52	109/86	29/18	Yes
Menezes 2008	Brazil (Brazilian)	Mixed	PCR-RFLP	HB	38/51	61/73	15/29	Yes
Moreira 2009	Brazil (Brazilian)	Mixed	PCR-RFLP	HB	55/43	95/72	15/14	Yes
Erciyas 2010	Turkey (Caucasian)	Non-smokers	PCR-SSP	PB	35/85	55/154	15/16	Yes
Scapoli 2011	Italy (Caucasian)	Unknown	PCR-MassARRAY	PB	122/246	1.19(0.81–1.74)^*^		Yes
Ebadian 2013	Iranian (Caucasian)	Non-smokers	PCR-RFLP	PB	58/60	74/88	42/32	Yes
Yang 2013	China (Asian)	Mixed	PCR-RFLP	HB	180/180	275/292	85/68	Yes
Özer Yücel 2015	Turkey (Caucasian)	Non-smokers	PCR-RFLP	HB	38/26	56/35	20/17	No

PB, population-based; HB, hospital-based; HWE, Hardy-Weinberg equilibrium; Mixed, smokers and non-smokers; *odds ratio with its 95% confidence interval.

**Table 2:** Results of overall and subgroups analyses.

Overall and subgroups	Number of Studies	Heterogeneity		Model	Meta-analysis	
		*p*	*I*^2^(%)		OR(95%CI)	*p* for OR
**A vs. G**	16	0.34	9.61	Fixed	1.23(1.04–1.44)	0.01
HWE (Yes)	14	0.32	12.35	Fixed	1.26(1.06–1.49)	0.01
HWE (No)	2	0.28	15.29	Fixed	1.04(0.65–1.65)	0.88
Population-based	11	0.56	0	Fixed	1.31(1.07–1.61)	0.01
Hospital-based	5	0.14	41.6	Fixed	1.10(0.85–1.43)	0.47
Smoking (Mixed)	7	0.45	0	Fixed	1.19(0.95–1.48)	0.13
Smoking (No)	6	0.09	47.68	Random	1.39(0.88–2.17)	0.15
Asian	3	0.74	0	Fixed	1.39(1.01–1.92)	0.04
Brazilian	2	0.62	0	Fixed	0.70(0.40–1.18)	0.18
Caucasian	9	0.3	15.37	Fixed	1.27(1.04–1.55)	0.02
Chileans	1	/	/	/	1.12(0.31–4.11)	0.86
**AA vs. GG**	15	0.14	36.5	Fixed	2.07(1.11–3.87)	0.02
HWE (Yes)	13	0.14	36.5	Fixed	2.07(1.11–3.87)	0.02
HWE (No)	2	–	–	–	–	–
Population-based	10	0.27	22.38	Fixed	2.73(1.01–7.35)	0.05
Hospital-based	5	0.04	75.6	Random	1.15(0.17–7.87)	0.89
Smoking (Mixed)	7	0.11	50.97	Random	2.18(0.56–8.44)	0.26
Smoking (No)	6	0.18	38.63	Fixed	1.95(0.64–6.00)	0.24
Asian	3	1	0	Fixed	2.71(1.09–6.73)	0.03
Brazilian	2	1	0	Fixed	0.37(0.07–2.02)	0.25
Caucasian	9	0.27	22.38	Fixed	2.73(1.01–7.35)	0.05
Chileans	1	/	/	/	–	–
**AG vs. GG**	15	0.22	20.56	Fixed	1.06(0.85–1.32)	0.62
HWE (Yes)	13	0.2	24.15	Fixed	1.06(0.83–1.34)	0.64
HWE (No)	2	0.18	44.51	Fixed	1.06(0.60–1.87)	0.85
Population-based	10	0.19	28.16	Fixed	1.15(0.85–1.55)	0.35
Hospital-based	5	0.35	9.53	Fixed	0.96(0.69–1.32)	0.79
Smoking (Mixed)	7	0.56	0	Fixed	1.01(0.77–1.32)	0.96
Smoking (No)	6	0.04	57.69	Random	1.21(0.81–1.82)	0.34
Asian	3	0.32	12.67	Fixed	1.14(0.77–1.69)	0.52
Brazilian	2	0.92	0	Fixed	0.75(0.40–1.42)	0.38
Caucasian	9	0.08	42.99	Random	1.06(0.70–1.59)	0.79
Chileans	1	/	/	/	1.14(0.29–4.45)	0.85
**AA vs. AG+GG**	15	0.13	37.95	Fixed	2.09(1.13–3.86)	0.02
HWE (Yes)	13	0.13	37.95	Fixed	2.09(1.13–3.86)	0.02
HWE (No)	2	–	–	–	–	–
Population-based	10	0.22	29.33	Fixed	2.65(0.99–7.04)	0.05
Hospital-based	5	0.05	73.93	Random	1.22(0.20–7.62)	0.83
Smoking (Mixed)	7	0.11	49.57	Fixed	2.20(1.05–4.61)	0.04
Smoking (No)	6	0.15	43.12	Fixed	1.86(0.62–5.63)	0.27
Asian	3	1	0	Fixed	2.75(1.12–6.75)	0.03
Brazilian	2	1	0	Fixed	0.42(0.08–2.19)	0.3
Caucasian	9	0.22	29.33	Fixed	2.65(0.99–7.04)	0.05
Chileans	1	/	/	/	–	–
**AA+AG vs. GG**	15	0.91	0	Fixed	1.06(0.85–1.31)	0.61
HWE (Yes)	13	0.93	0	Fixed	1.06(0.84–1.33)	0.64
HWE (No)	2	0.18	44.51	Fixed	1.06(0.60–1.87)	0.85
Population-based	10	0.99	0	Fixed	1.08(0.80–1.45)	0.62
Hospital-based	5	0.24	26.65	Fixed	1.03(0.76–1.41)	0.84
Smoking (Mixed)	7	0.58	0	Fixed	1.10(0.85–1.43)	0.45
Smoking (No)	6	0.83	0	Fixed	0.98(0.65–1.46)	0.91
Asian	3	0.52	0	Fixed	1.29(0.89–1.88)	0.18
Brazilian	2	0.74	0	Fixed	0.70(0.38–1.30)	0.26
Caucasian	9	0.92	0	Fixed	1.02(0.76–1.37)	0.89
Chileans	1	/	/	Fixed	1.14(0.29–4.45)	0.85

OR, odds ratio; CI, confidence interval; HWE, Hardy-Weinberg equilibrium; –, the AA frequency = 0; /, only one study included.
