# Supplementary material for: Tumor necrosis factor-α G-308A (rs1800629) polymorphism and aggressive periodontitis susceptibility: a meta-analysis of 16 case-control studies
Source: Sci Rep. 2016 Jan 11;6:19099. doi: 10.1038/srep19099 (PMC4707537; doi:10.1038/srep19099)

Tumor necrosis factor-α G-308A (rs1800629) polymorphism and aggressive periodontitis susceptibility: a meta-analysis of 16 case-control studies

Xue-Mei Wei[1*], Yong-Ji Chen[2*], Lan Wu[2*], Li-Jun Cui[3], Ding-Wei Hu[1], Xian-Tao Zeng[2,3]

1. Department of Nursing, Affiliated Hospital of North Sichuan Medical College, Nanchong 637000, P.R. China;

2. Department of Stomatology, Taihe Hospital, Hubei University of Medicine, Shiyan 442000, Hubei Province, China;

3. Department of Vascular Surgery, Affiliated Hospital of North Sichuan Medical College, Nanchong 637000, P.R. China;

4. Center for Evidence-Based and Translational Medicine, Zhongnan Hospital of Wuhan University, Wuhan 430071, China

*These authors contributed equally to this work.

Running title: TNF-α G-308A polymorphism and aggressive periodontitis.

Correspondence to: Xian-Tao Zeng, Center for Evidence-Based and Translational Medicine, Zhongnan Hospital of Wuhan University, 169 Donghu Road, Wuchang District, Wuhan 430071, Hubei Province, P.R. China. E-mail: zengxiantao1128@163.com; Tel: +86 027 6781 2817, Fax: +86 027 6781 2817.

Supplementary Fig. S1 Forest plot of overall analysis in AA vs. GG genetic model

**Study name**	**Statistics for each study**					**OR and 95% CI (Fixed)**
	OR	LI	UI	Z-Value	p-Value	
Sakellari 2006	9.64	0.45	206.39	1.45	0.15	
de Freitas 2007	0.33	0.04	2.93	-0.99	0.32	
Guzeldemir 2008	7.00	0.32	155.03	1.23	0.22	
Schulz 2008	8.34	0.45	155.99	1.42	0.16	
Menezes 2008	0.37	0.07	2.02	-1.14	0.25	
Erciyas 2010	0.70	0.03	15.11	-0.23	0.82	
Ebadian 2013	5.00	0.97	25.77	1.92	0.05	
Yang 2013	2.71	1.09	6.73	2.15	0.03	
**Total**	2.07	1.11	3.87	2.29	0.02	

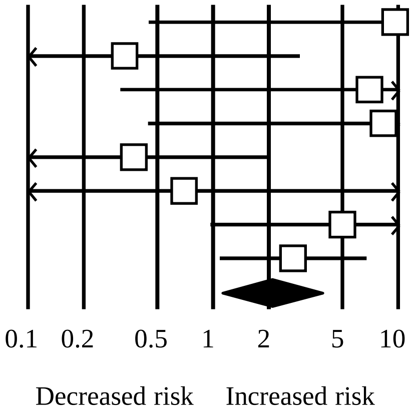

Heterogeneity: I^2 = 36.50%, p = 0.14

OR = Odds ratio; LI = Lower limit; UI = Upper limit; CI = Confidence interval

Supplementary Fig. S2 Forest plot of overall analysis in AA vs. AG+GG genetic model

**Study name**	**Statistics for each study**				
	OR	LI	UI	Z-Value	p-Value
Sakellari 2006	10.17	0.48	216.33	1.49	0.14
de Freitas 2007	0.34	0.04	2.95	-0.97	0.33
Guzeldemir 2008	8.08	0.37	177.23	1.33	0.18
Schulz 2008	8.95	0.48	165.66	1.47	0.14
Menezes 2008	0.42	0.08	2.19	-1.03	0.30
Erciyas 2010	0.47	0.02	10.05	-0.48	0.63
Ebadian 2013	4.64	0.94	22.87	1.89	0.06
Yang 2013	2.75	1.12	6.75	2.20	0.03
**Total**	2.09	1.13	3.86	2.35	0.02

OR and 95% CI (Fixed)

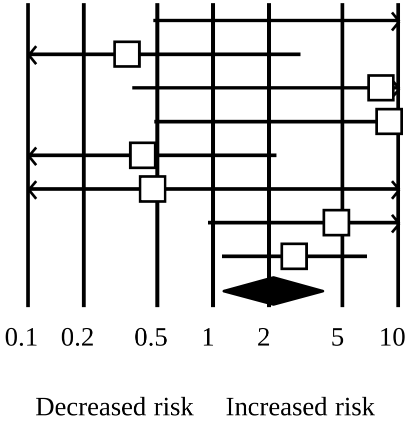

Heterogeneity: I^2 = 37.95%, p = 0.13

OR = Odds ratio; LI = Lower limit; UI = Upper limit; CI = Confidence interval

Supplementary Fig. S3 Forest plot of overall analysis in AG vs. GG genetic model

**Study name**	**Statistics for each study**				
	OR	LI	UI	Z-Value	p-Value
Endo 2001	1.14	0.20	6.44	0.14	0.89
Shapira 2001	0.66	0.14	3.02	-0.54	0.59
Pérez 2004	1.14	0.29	4.45	0.18	0.85
Brett 2005	1.37	0.69	2.73	0.90	0.37
Sakellari 2006	0.81	0.35	1.88	-0.49	0.62
Zhu 2007	1.99	0.87	4.55	1.63	0.10
de Freitas 2007	0.92	0.40	2.14	-0.19	0.85
Guzeldemir 2008	0.40	0.07	2.19	-1.05	0.29
Schulz 2008	0.80	0.36	1.75	-0.57	0.57
Menezes 2008	0.72	0.29	1.84	-0.68	0.50
Moreira 2009	0.78	0.33	1.86	-0.57	0.57
Erciyas 2010	4.44	1.79	10.99	3.22	0.00
Ebadian 2013	1.16	0.54	2.47	0.39	0.70
Yang 2013	0.96	0.60	1.52	-0.19	0.85
Ozer Yücel 2015	0.59	0.21	1.65	-1.01	0.31
**Total**	1.06	0.85	1.32	0.50	0.62

OR and 95% CI (Fixed)

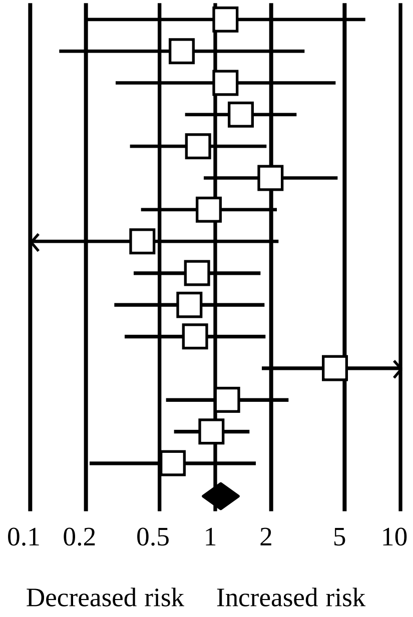

Heterogeneity: I^2 = 20.56%, p = 0.22

OR = Odds ratio; LI = Lower limit; UI = Upper limit; CI = Confidence interval

Supplementary Fig. S4 Forest plot of overall analysis in AA+AG vs. GG genetic model

**Study name**	OR	LI	UI	Z-Value	p-Value
Endo 2001	1.14	0.20	6.44	0.14	0.89
Shapira 2001	0.66	0.14	3.02	-0.54	0.59
Pérez 2004	1.14	0.29	4.45	0.18	0.85
Brett 2005	1.37	0.69	2.73	0.90	0.37
Sakellari 2006	0.97	0.43	2.18	-0.07	0.94
Zhu 2007	1.99	0.87	4.55	1.63	0.10
de Freitas 2007	0.81	0.36	1.83	-0.50	0.62
Guzeldemir 2008	0.81	0.20	3.20	-0.31	0.76
Schulz 2008	1.01	0.47	2.15	0.02	0.98
Menezes 2008	0.63	0.27	1.51	-1.03	0.30
Moreira 2009	0.78	0.33	1.86	-0.57	0.57
Erciyas 2010	1.27	0.41	3.95	0.41	0.68
Ebadian 2013	1.33	0.64	2.77	0.77	0.44
Yang 2013	1.16	0.75	1.78	0.66	0.51
Ozer Yücel 2015	0.59	0.21	1.65	-1.01	0.31
**Total**	1.06	0.85	1.31	0.50	0.61

Columns OR, LI, UI, Z-Value and p-Value are headed "**Statistics for each study**".

Heterogeneity: I^2 = 0%, p = 0.91

OR and 95% CI (Fixed)

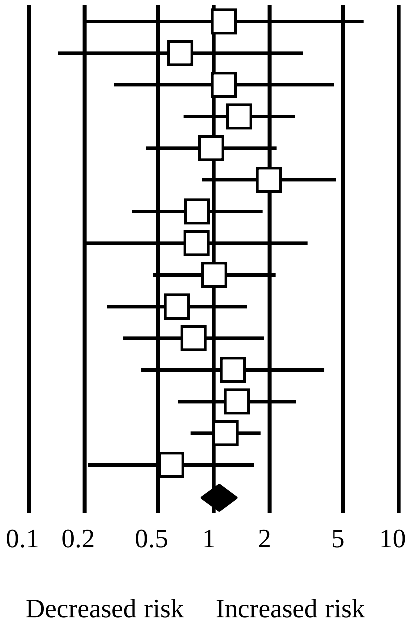

OR = Odds ratio; LI = Lower limit; UI = Upper limit; CI = Confidence interval

Supplementary Fig. S5 Forest plot of meta-regression analysis in A vs. G genetic model based on smoking status

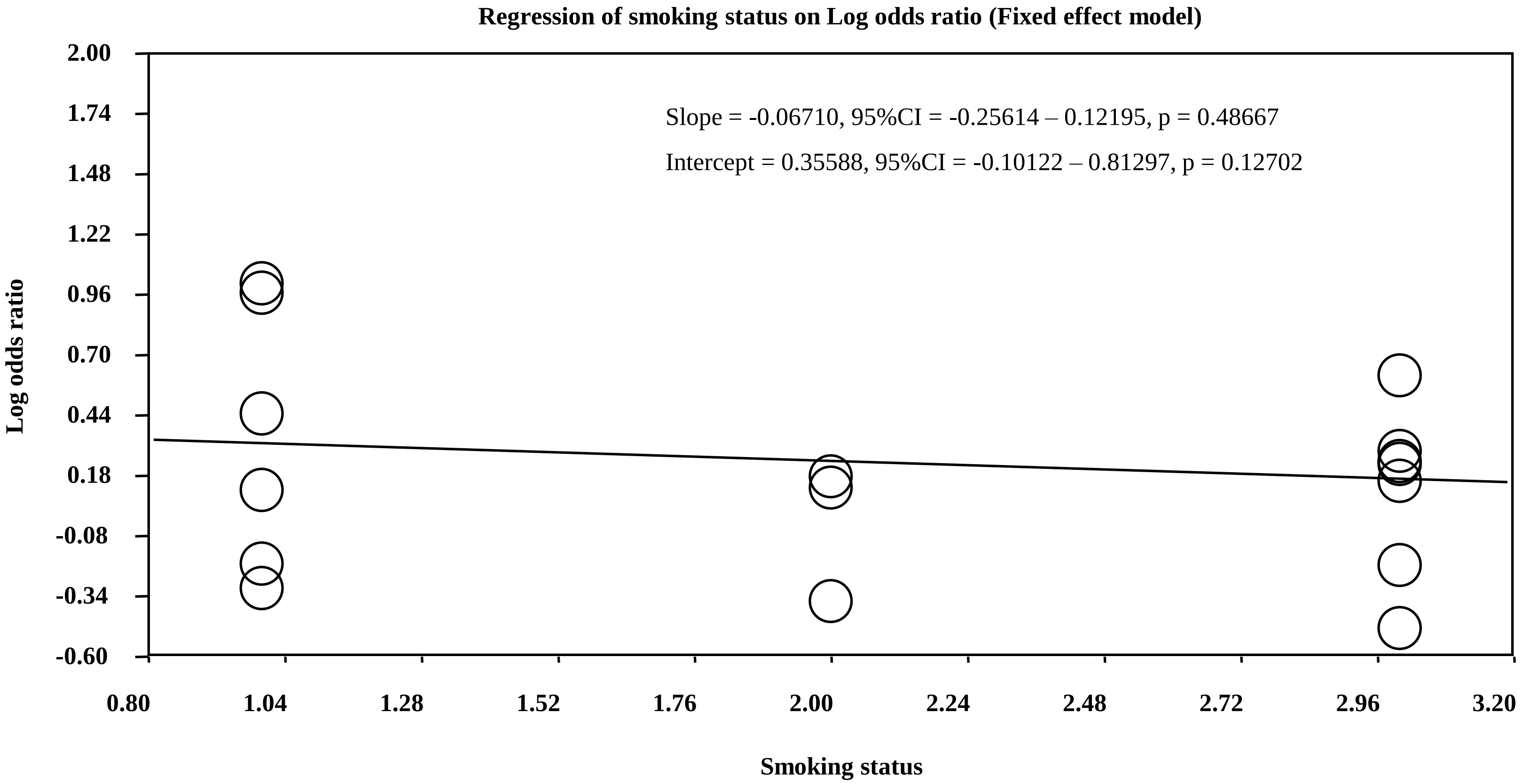

Supplement: Supplementary Information [file srep19099-s1.pdf]
